# Population-based monitoring of HIV drug resistance early warning indicators in Uganda: A nationally representative survey following revised WHO recommendations

**DOI:** 10.1371/journal.pone.0230451

**Published:** 2020-04-14

**Authors:** Juliet Asio, Christine Watera, Norah Namuwenge, Wilford Kirungi, Joshua Musinguzi, Kaggwa Mugagga, Ronald Busobozi, Bridget Jolly Tusiime, Tom Lutalo, Edward Katongole Mbidde, Pontiano Kaleebu, Uganda HIV Drug Resistance Technical Working Group

**Affiliations:** 1 Uganda Virus Research Institute, Entebbe, Uganda; 2 Uganda AIDS Control Programme, Ministry of Health, Kampala, Uganda; 3 Malaria and Non Communicable Diseases, World Health Organisation-Uganda Country office, Kampala, Uganda; 4 Basic Sciences Programme, Medical Research Council (MRC)/Uganda Virus Research Institute (UVRI) and London School of Hygiene and Tropical Medicine (LSHTM) Uganda Research Unit, Entebbe, Uganda; 5 Department of Infection Biology, London School of Hygiene and Tropical Medicine, London, United Kingdom; Albert Einstein College of Medicine, UNITED STATES

## Abstract

**Introduction:**

With the scale-up of antiretroviral therapy (ART) there is a need to monitor programme performance to maximize ART efficacy and to prevent emergence of HIV drug resistance (HIVDR). In keeping with the elements of the World Health Organisation (WHO) guidance we carried out a nationally representative assessment of early warning indicators (EWI) at 304 randomly selected ART service outlets in Uganda.

**Methods:**

Retrospective patient data was extracted for the six EWIs for HIVDR including; on-time antiretroviral (ARV) drug pick-up, patient retention on ART at 12 months, ART dispensing practices, ARV drug stock-outs, viral load suppression (VLS) and viral load (VL) testing completion. Point prevalence for each clinic and national aggregate prevalence with 95% confidence intervals (CI) for all clinics were estimated and facility performances were computed and association between EWIs and programmatic factors assessed using Fisher’s Exact Test.

**Results:**

Facilities meeting the EWI targets: *on-time pill pick-up* was 9.5%, more facilities in the north met this target (p = 0.040). *Retention on ART at 12 months* was 24.1%, facilities in Kampala region (p<0.001) and Specialized ART clinics (p = 0.01) performed better in this indicator. *Pharmacy stock-outs* was 33.6%, with more facilities in Kampala (p<0.001), specialized ART clinics (p<0.001) and private-for-profit (p<0.001) meeting this target. *Dispensing practices* was met by 100% of the facilities. VLS was met by 49.2% and 50.8% of facilities met VL completion target with facilities in central region performing better (p<0.001). National prevalence for the EWIs was: *on-time pill pick-up* 63.3% (CI: 58.9–67.8); *retention on ART at 12 months* 69.9% (CI: 63.8–76.0); *dispensing practices* 100.0%; *VLS* 85.2% (CI: 81.8–88.5) and *VL completion*, 60.7% (CI: 56.9–64.6).

**Conclusion:**

Dispensing practices in all facilities were in line with the national guidelines however, there still remains a challenge to long-term ART programmatic success in monitoring patient response to treatment, and maintaining patients on ART without interruptions arising due to poor patient adherence and as a consequence of ARV supply interruption. It is therefore of high importance that the national ART program ensures intensified follow-up for patients, ensuring uninterrupted supply of ARV drugs and increasing VL monitoring at treatment centres, in order to improve patient outcomes and avert preventable HIVDR

## Introduction

Over the last decade, the global AIDS prevention and control strategy has registered significant progress towards reducing AIDS-related deaths. The global scale-up of combination antiretroviral therapy (ART) under the public health approach of standardized and simplified regimens and the implementation of the WHO test and start strategy, has led to increased access to treatment for millions of people, a reduction in new infections as well as HIV-associated morbidity and mortality [1, 2]. In 2018, 940,000 [670 000–1.3 million] people died from AIDS-related illnesses worldwide, compared to 1.4 million [1 million–2 million] in 2010 and 1.9 million [1.4 million–2.7 million] in 2004 [3], registering a 50% reduction. In sub-Saharan Africa alone, the region most affected by HIV/AIDS; high level political commitment and substantial international funding have led to increased access to ART [4]. By the end of 2017, there were 25.7 million people living with HIV in sub Saharan Africa [3], and about 60% of these were receiving ART compared, to 5.0 million (21.3%) in 2010 [5].

The global roll-out of ART has inevitably been accompanied by the emergence of HIV drug resistance (HIVDR), as has been observed in countries where ART has been available for longer periods [6]. The emergence of HIVDR could reduce the efficacy of HIV prevention strategies and lead to the eventual failure of ART programmes [6]. In order to minimize preventable HIVDR at programme level, routine monitoring of programme performance and treatment outcomes is vital. The WHO developed a Global Action Plan on HIV Drug Resistance that recommends national HIVDR prevention strategies to be developed and integrated into all ART scale-up initiatives [7]. This strategy involves (i) prevention and response through implementation of high impact interventions, ii) monitoring and surveillance through periodic surveys, iii) carrying out relevant and innovative research iv) supporting laboratory capacity by expanding viral load (VL) and HIVDR testing and v) ensuring governance and enabling mechanisms to support HIVDR activities. The HIVDR Monitoring and Surveillance Strategy on prevention, monitoring and response to HIVDR guides national programs in obtaining population-level data on emergence of HIVDR in different populations. Monitoring of HIVDR early warning indicators (EWI) provides an alternative method to assess the risk of HIVDR in settings where HIVDR testing at the patient-level is not available, and also provides a means of timely identification of clinics with suboptimal performance which helps to target appropriate interventions that can optimize ART programme performance. These indicators include: appropriate prescribing practices, medication adherence, strengthening antiretroviral (ARV) logistics management, regular VL monitoring and systematic collection of strategic information to track emergence of HIV drug resistant strains.

In keeping with one of the elements of the WHO recommendation of monitoring EWIs of HIVDR, Uganda has previously carried out 4 rounds of assessment at selected ART service outlets. These included 41 facilities in 2007, 75 facilities in 2008, 95 facilities in 2012 and 96 facilities during 2014 [8]. The results showed that prescribing practices in most facilities were in line with national guidelines. However, clinic review appointment keeping, on-time pill pick up and continuity of drug supply manifested the most profound weaknesses at the programme level. Recommendations from these surveys have resulted into new policy formulations and influenced ART programme practice. These include; the roll out of standardized and harmonized electronic medical records system integrated with EWIs to ART facilities, increased funding towards HIV drugs in Uganda, ARV distribution rationalized to three warehouses compared to one in the past, and revision of national the VL testing algorithm to provide for 12 monthly VL monitoring for newly initiating individuals.

This present survey is therefore, is a follow-on of the previous rounds and is the first nationally representative assessment of EWIs for HIVDR in a sample of 304 facilities across the country, following revised WHO guidance. The main purpose of this survey was to determine point prevalence at clinic level and a nationally representative prevalence estimate of EWIs for HIVDR. We also determined association between EWIs and programmatic factors.

## Methods

### Study setting

Uganda has been rolling out provision of ART since 2004, starting under the WHO/UNAIDS 3 X 5 initiative [9]. The Ministry of Health accredits facilities for provision of ART, based on their ability to offer services in accordance with the minimum national standards. Currently, there are about 1800 facilities accredited to provide ART countrywide. These facilities are at various levels, from primary health centres, hospitals and specialized ART clinics; some of which programmes provide community and home-based ART services. Uganda adopted the 2015 WHO guidelines that provide for treatment for all identified PLHIV [10]. The country also adopted VL testing as the preferred method for ART monitoring. Between 2014 and 2015 VL testing was done at six months after ART initiation, then at 18 months and every 12 months thereafter, however in 2016 VL testing guidelines were changed to every 6 months for children and adolescents; while in adults 6 months after ART initiation, then at 12 months, and every 12 months thereafter. Uganda has implemented the WHO recommended VL monitoring program for all patients on ART since 2014 and the VL coverage has increased from 47% in 2016 to 85% in 2018 [11], and VLS is considered as <1000 copies/μL. By December 2018, it was estimated that there were 1,324,660 people living with HIV in Uganda and 1,167,025 (88%) of these were on treatment. Among those on ART, 1,140,550 were eligible for VL testing and 1,001,595 (88%) of these, had VL testing done. Viral load suppression was found in 892,393 (89%) of those tested [11]. Viral load testing for all sites in the country is done at the Central Public Health Laboratories (CPHL) using the National Sample *and* Results Transport Network for both DBS and plasma *Samples*. DBS samples are shipped at room temperature whereas plasma specimens are transported in liquid nitrogen tanks to CPHL. VL testing was done at CPHL using a DBS-VL protocol using the Abbott RealTime HIV-1 Assay Kit (Abbott Molecular Diagnostics, Des Plaines, IL) and plasma using COBAS^®^ AmpliPrep/COBAS^®^ TaqMan^®^ HIV-1 Test, v2.0 kit (Roche Molecular System, Inc. Pleasanton, CA).

### Study design

This was a retrospective cross-sectional survey conducted using a stratified two-stage cluster design among 304 randomly selected ART sites in Uganda. The design enabled inclusion of facilities from all geographical regions, all levels of ART service delivery, as well as different ART service delivery modes; including government public clinics, non-governmental organizations (NGOs), some community-based organisations, faith-based organizations and private for-profit clinics.

### Population and sampling

Selection of patient records was done using a two-stage process. In the first stage, 309 facilities were selected from among all Ministry of Health, AIDS Control Programme (MoH/ACP) accredited ART facilities in the country that had dispensed ART for at least 2 years. The facilities were stratified by geographic region, and by level of service delivery and facilities were sampled with randomly with the probability of inclusion proportional to the number of sites in each stratum.

The country was divided into 5 geographical regions (strata) by combining areas used in the Uganda Population-based HIV Impact Assessment (UPHIA) [12] survey to enable stratification. The geographical areas included Central (Central 1 and Central 2), East (East Central, Mid-East and North-East), Kampala (stand-alone, is the capital city of Uganda), North (Mid Northern and West Nile) and West (Mid-Western and South-Western).

The second stage of stratification was based on the level of ART service delivery. Uganda's health system is divided into national and district-based levels. At the national level, ART is provided at the national referral hospitals and at regional referral hospitals; whereas at district level, ART provision is at district hospitals, specialized ART clinics, and at health centres. Health are located at both sub-county and county levels and they serve a population of at least 10,000 people. Facilities are either government-owned, privately owned or are private-not-for-profit. The not-for-profit providers are run on a national and local basis and over 70% are faith-based.

The sample size was determined using Epi Info version 3.4.1 for estimation of sample size for a proportion. The proportion of facilities that did not meet the recommended WHO targets for any of the EWI from the 2014 round of assessment, was used to estimate the sample size. Based on precision around the estimate, from the approximately 1600 otherwise eligible facilities, the sample size was calculated using the formula: $n0=\frac{Z^{2}p(1-p)}{e^{2}}\times (1+\frac{r}{100})$ and applying the correction factor $n=\frac{n_{0}}{1+\frac{n_{0}-1}{N}}.$ where: n_0_ = sample size before the application of the correction factor, n = sample size after applying the correction factor, p = HIVDR EWI-specific prevalence based on the 2014 assessment, d = precision (5%), Z_1- ⍺/2_ = desired level of confidence, α = 0.05 (1.96), N = Number of facilities that were offering ART services between December 2014 and November 2015. A sample of 309 facilities was required for this survey.

The same formula was used to determine the sample size for patient records for each indicator at facilities, where N for facility, would be the eligible number of patient records for each indicator.

### Sampling reference periods

The EWIs are divided into cross sectional indicators, (on-time pill pick-up, and dispensing practices) and indicators based on a 12-month reporting period (retention, stock-outs, viral load suppression (VLS) and VL completion).

The timeline for sampling of patients was based around a 12 months reporting period required for retention, VLS and drug stock-out indicator. Individuals who initiated ART in the period from 1^st^ July 2015 to 30^th^ June 2016, constituted outcomes for retention, VLS at 12 months after ART initiation and VL completion. Data for pharmacy stock-outs was obtained for the period from 1^st^ July 2016 to 30^th^ June 2017 from facility based pharmacy and store stock cards.

Assessment of on-time pill pick-up was done by abstracting patient data on the baseline ART pick-up and one subsequent pick-up, for the period from 1^st^ July 2016 to 30^th^ June 2017. Recruitment of patient records was consecutive until the required sample size was reached.

### Data collection

The WHO site profile data collection tool was used to collect facility-based variables including: type of ART programme in the facility, ART patient records used at facility, client volume, ART dispensing staff and days of when ART provision.

Patient and pharmacy data was abstracted into standardized data extraction tools adapted from the WHO ResNet excel-based tool [13]. The ART register, chronic AIDS care/ ART cards (paper-based or electronic) and viral load registers constituted the primary source of data for abstraction of patient records at each facility. Pharmacy data was obtained from pharmacy registers and ARV dispensing logs and store stock cards.

### Data management and analysis

Site profile data was entered into a Microsoft Access database. The electronic tools are programmed to determine for each facility whether they meet the requirements for each indicator. For each facility, a point prevalence (numerator and denominator) was obtained for each EWI, and this was compared to EWI-specific thresholds to determine the appropriate colour–coded classification; green for excellent performance, amber for fair performance and red for poor performance, and grey if more than 70% of the data is missing at the clinic.

For national level reporting, all the clinic-level scores for each indicator was aggregated into a single Ms Excel file using the WHO EWI aggregator tool. This tool also extracts data required for weighting each indicator required for calculating nationally representative EWI prevalence estimates.

Patient level data was extracted from the electronic tools to create separate datasets for each indicator. These datasets were then exported to Stata for analysis. Each dataset for the EWIs were merged with the site profile and aggregated data using facility as the unique identifier, to obtain a single master file for analysis for each EWI.

At national level, the proportion of clinics that scored green, amber or red for each indicator, was determined. In addition, nationally representative weighted prevalence estimates and 95% confidence intervals for each EWI (excluding drug stock-outs) was calculated after weighting the clinic data. This was done using two-stage clustered survey design that accounted for the survey stratification, clustering and survey weights using the svyset function Weights accounted for *unequal selection probabilities*, for both selection of clinics and selection of patients. Stratification was done to reduce sampling variation to account for stratification by geographical region during selection of clinics. Clustering accounted for the two clustering units, that is;- patients and clinics. Associations between different programmatic factors and performance of EWI were also determined using Chi-square tests. All statistical analyses were performed using STATA version 14 (StataCorp LP, Texas, USA).

### EWI definitions

#### On-time pill pick-up

Percentage of patients that pick-up ART no more than two days late at the first pick-up after the baseline pick-up (performance target: excellent > 90%, fair 80–90% and poor <80). Numerator: Number of patients picking up their ART “on-time” at the first drug pick-up after baseline pick-up. Denominator: number of patients who picked up ARV drugs on or after the designated EWI sample start date.

#### Patient retention on ART

Percentage of patients known to be alive and on treatment 12 months after initiation of ART (performance target: excellent >85%, fair 75–85% and poor <75%). Numerator: Number of patients who are still alive and on ART 12 months after initiating treatment. Denominator: Total number of patients who initiated ART who were expected to achieve 12-month outcomes within the reporting period, including those who have died since starting therapy, those who have stopped therapy, and those recorded as lost to follow-up at month 12.

#### Pharmacy stock outs

Percentage of months with no day(s) of stock-out of any routinely dispensed ARV drug (performance target: excellent 0%, poor >0%). Numerator: number of months in the designated year in which there were no stock-out days of any ARV drug routinely used at the site. Denominator: 12 months.

#### ARV drug dispensing practices

Percentage of patients prescribed or picking up mono or dual ARV therapy (performance target: excellent 0% and poor > 0%). Numerator: number of patients who pick-up from the pharmacy, a regimen consisting of one or two ARVs. Denominator: number of patients picking up ART on or after the designated EWI sample start date.

#### Virological load suppression

Percentage of patients receiving ART at the site after the first 12 months of ART whose VL is <1000 copies/ml (performance target: excellent > 90%, fair 80–90%, poor <80%). Numerator: number of patients receiving ART at the site after the first 12 months of ART whose viral load is <1000 copies/ml. Denominator: number of patients at the site who by national policy should have had a VL performed 12 months after ART initiation.

#### Viral load completion

Percentage of patients with a 12-month VL test result available (performance target: excellent ≥70%, poor <70%). Numerator: Number of patients alive and on ART 12 months after treatment initiation who have a VL test result available. Denominator: Number of patients alive and on ART 12 months after treatment initiation, who are therefore, consistent with the policy, expected to have a VL test result available in the primary medical record.

### Ethical considerations

Ethical approval was obtained from the Uganda Virus Research Institute Science and Ethics Committee, Uganda National Council for Science and Technology and the office of the Associate Director for Science office at the Centers for Disease Control and Prevention. No contact with patients occurred during this surveys, and secondary data collection was done using patient records and facility registers. Patient records were anonymised and no personal identifiable information of the patients was collected.

## Results

This assessment was conducted between October 2017 and March 2018. Three hundred and nine facilities were chosen for this survey, however five facilities were excluded from this report because two were in hard-to reach areas, and data was not collected; and the other three facilities did not have readily available patient records.

### Characteristics of ART facilities

Regional distribution showed that the majority of facilities were from west (28.0%) and central (26.3%), which reflects the national paradigm (Table 1). At facility level, most of the sites were health centres 238 (78.3%), including health centres IIIs and IVs, 46 (15.1%) were hospitals—including 39 district/general hospitals, 6 Regional Referral Hospitals, 1 National Referral Hospital; there were 14 (4.6%) Specialized ART clinics and only 6 (2.0%) private clinics. Government aided facilities constituted 81.9% of the sample, the others were either private for profit, or non-profit facilities; most of which are faith-based.

**Table 1:** Facility characteristics and performance in WHO early warning indicator targets.

Programmatic Characteristics	Facility counts n(%)	HIVDR Early Warning Indicators					
		On-time Pill pick-up	Retention on ART at 12 months	Dispensing practices	Drug Stock outs	Viral load suppression	Viral load completion
		(Target>90%)	(Target>85%)	(Target = 100%)	(Target = 0%)	(Target≥90%)	(Target≥70%)
**Geographical Region**							
Central	80(26.3)	13(16.3)	15(18.8)	80(100.0)	37(46.3)	39(48.8)	60(75.0)
East	73(24.0)	7(9.6)	11(15.1)	73(100.0)	15(20.5)	29(39.7)	57(78.1)
Kampala	19(6.2)	0(0.0)	9(47.4)	19(100.0)	13(68.4)	9(47.4)	8(42.1)
North	47(15.5)	6(12.8)	5(10.6)	47(100.0)	14(29.8)	26(55.3)	3(6.4)
West	85(28.0)	3(3.5)	34(40.0)	85(100.0)	23(27.1)	45(52.9)	28(32.9)
*P*-value		0.036	<0.0001	1.000	<0.0001	0.433	<0.0001
**Level of Facility**							
Health Centre	238(78.3)	20(8.4)	50(21.0)	238(100.0)	66(27.7)	11(4.6)	124(52.1)
Hospital	46(15.1)	7(15.2)	13(28.3)	46(100.0)	23(50.0)	25(54.3)	22(47.8)
Private clinic	6(2.0)	0(0.0)	3(50.0)	6(100.0)	3(50.0)	2(33.3)	3(50.0)
Specialized ART clinic	14(4.6)	2(14.3)	8(57.1)	14(100.0)	10(71.4)	7(50.0)	7(50.0)
*P*-value		0.381	0.007	1.000	<0.0001	0.747	0.954
**Ownership**							
Government	249(81.9)	22(8.8)	54(21.7)	249(100.0)	74(29.7)	118(47.4)	132(53.0)
Private For Profit	11(3.6)	0(0.0)	5(45.5)	11(100.0)	9(45.5)	6(54.5)	5(45.5)
Private Not For Profit	44(14.5)	7(15.9)	15(34.1)	44(100.0)	19(43.2)	24(54.5)	19(43.2)
*P*-value		0.185	0.053	1.000	0.001	0.630	0.433

Among the selected facilities, the cumulative number of patients ever on ART was 632,533 of which 286,772 were still active on ART at the time of conducting the survey. The total number of patients who had initiated ART 12 months prior to the survey reference period of 1^st^ July 2015 to 30^th^ June 2016 was 47,783. There were 2,514 ART care providers at the selected facilities, giving a staff-patient ratio of 1:114. The ART care providers consisted of physicians, nurses, laboratory personnel, counselors and clinical officers. The total number of pharmacy and/or dispensing staff was 1,419, giving a pharmacist to patient ratio of 1:202.

There were different cohorts of patients assessed for each indicator. The total number of patients assessed for the indicators of on-time pill pick-up and for dispensing practices was 162,441. Retention in care was assessed from 104,903 patient records; viral load suppression from 22,896 patient records VLS and VL completion from 37,830 patient records.

### On-time pill pick-up

**Fig 1:**
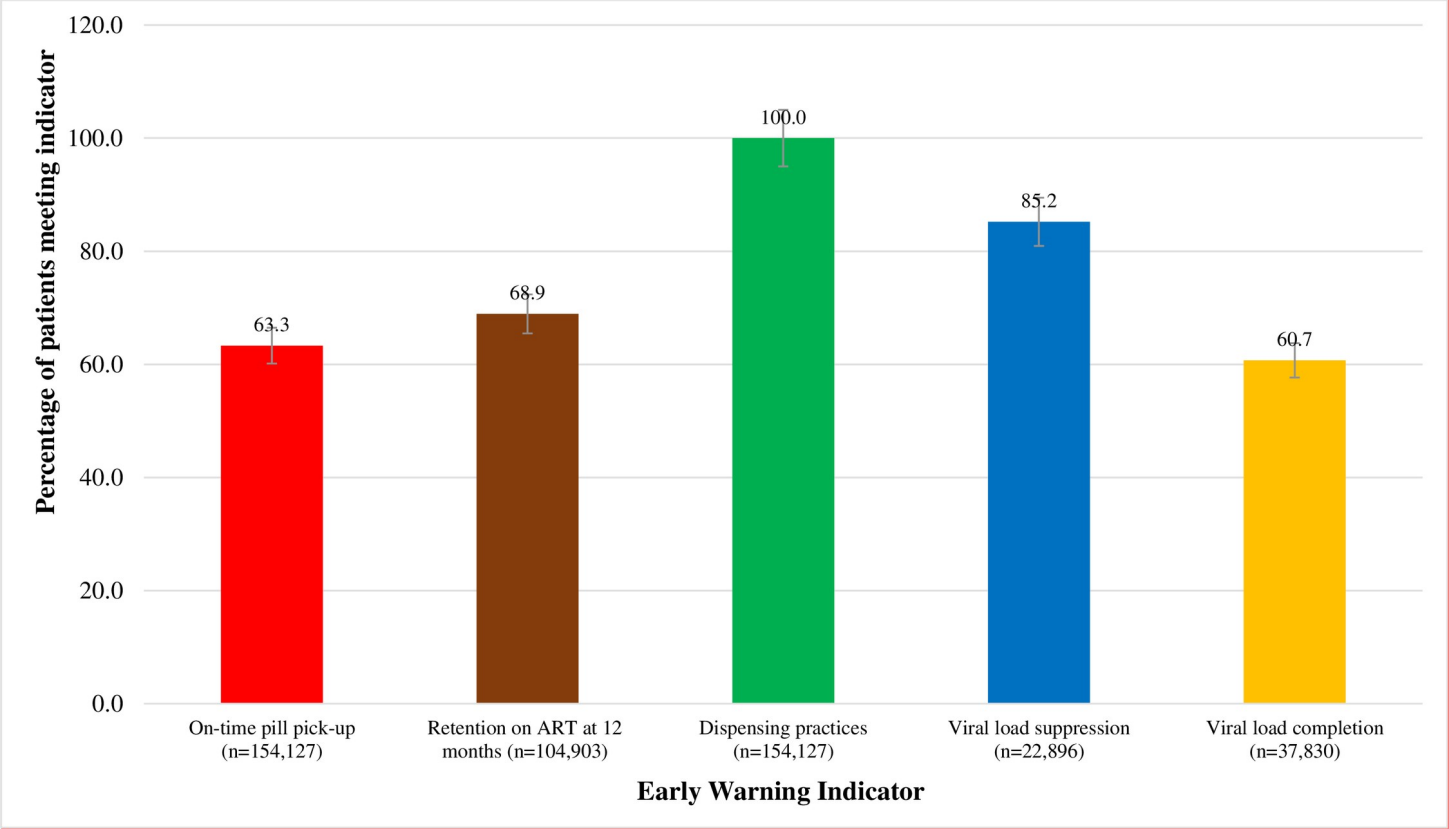
National aggregate prevalence for WHO early warning indicators.

Of the 162,441 patient records that were eligible analysed for this indicator, 8,314 (5.1%) patient records were excluded because they did not have the subsequent pill pick-up date. Of the, 154,127 patient records considered, 95,334 (63.3% CI: 58.9–67.8 had picked up their pills on-time. This represents the proportion of patients in Uganda who pick-up their drugs on time, for the reference period (Fig 1).

At facility level, 29 (9.5%) had good performance, 50 (16.5%) had fair performance and 225 (74.0%) had poor performance (Table 2). Performance by region showed that facilities from the north met the target for this indicator compared to other regions. The difference in proportion of facilities meeting the target across the regions was statistically significant (*P* = 0.04). Kampala region had none of the facilities meeting the target (Table 1). Neither facility level nor ownership had a significant effect on the performance of the facility on on-time pill pick-up.

**Table 2:** Performance of ART facilities in WHO early warning indicators for HIVDR in Uganda.

WHO-recommended EWIs of HIVDR	EWI Target (green: good; amber: fair; red: poor)	ART Facilities meeting EWI Target (%)
**On time ARV-drug pick up**; % of patients that pick-up ART no more than two days late at the first drug pickup after a defined baseline pick-up	**Green:>90**	29 (9.5)
	**Amber:80–90**	50 (16.5)
	**Red:<80**	225 (74.0)
**Retention on ART at 12 months**; % of patients retained on ART 12 months after ART initiation^i^	**Green:>85**	73 (24.1)
	**Amber:75–85**	69 (22.8)
	**Red:<75**	161 (53.1)
**Pharmacy stock outs**; % of months with any day(s) of stock-out of any routinely dispensed ARV drug	**Green:100**	102 (33.6)
	**Red:<100**	202 (66.4)
**Dispensing Practices**; % of patients prescribed or picking up mono or dual ARV therapy	**Green:0**	304 (100.0)
	**Red:>0**	0 (0.0)
**Viral load suppression**; % of patients with viral load <1000 copies/mL 12 months after ART initiation^ii^	**Green: ≥90**	146 (49.2)
	**Amber:80-<90**	108 (36.3)
	**Red:<80**	43 (14.5)
**Viral load completion**; % of patients with a 12-month viral load test result available^ii^	**Green: ≥70**	151 (50.8)
	**Red:<70**	146 (49.2)

^i^ one result missing

^ii^ 7 results missing, Green (good performance) is considered as met target, amber and red mean that facility did not meet the target

### Retention on ART at 12 months

Out of the 104,903 clients who initiated ART between 1 July 2015 to 30 June 2016 from the selected facilities, 72,294 (69.9%, CI: 63.8–76.0%) were alive and on ART 12 months later.

Facility performance showed that 73 (24.1%) of the facilities retained their clients on ART 12 months after initiation, 69 (22.8%) facilities had fair performance and 161 (53.1%) performed poorly in this indicator. More facilities in Kampala met this indicator target (*P*<0.0001), compared to other regions (Table 1). Specialized ART clinics and private clinics were more likely to meet this indicator target (*P* = 0.007), and health centres were the least likely to meet this target (21.0%), (Table 1).

### Pharmacy stock outs

During this assessment, 102 (33.6%) of the facilities met this indicator target. Kampala had the highest proportion of facilities (n = 13, 68.4%), meeting this indicator target compared to other regions, and this difference was statistically significant (*P*<0.0001) (Table 1). More specialized clinics (71.4%) met this target, compared to other facility levels (p<0.0001). Privately-owned facilities compared to government-owned facilities (*P* = 0.001) met the target for pharmacy stock-outs.

### Dispensing practices

All facilities assessed scored 100% in this indicator, having none of patients picking up mono or dual ARV therapy.

### Viral load suppression

There were 37,830 patients that were eligible for VL testing during the survey period in the selected facilities. Out of these, VL results were available for 22,896 (60.5%) patients; and among these, 19,488 (85.2% CI: 81.8–88.5) patients had VLS.

At facility level, 108 (36.4%) had good performance, 108 (36.4%) had fair performance and 43 (14.5%) had poor performance. Seven facilities did not have data on VL, and thus were categorised as grey. There was no significant difference in performance of facilities across the different characteristics for this indicator (Table 1).

### Viral load completion

There were 37,830 patient records that were analysed for this indicator. Out of these, 22,896 had VL results, giving a prevalence of 60.7% (CI: 56.9–64.6). At facility level, 29 (9.5%) had excellent performance, 50 (16.4%) had fair performance and 225 (74.0%) had poor performance. Facilities from the east region performed better in this indicator (78.1%) compared to other regions, whereas those in the north had the least proportion meeting this indicator target (6.4%, p<0.0001). There was no statistically significant difference in the performance of facilities by level nor by ownership.

### Overall facility performance

Generally only one (0.3%) facility met all the six EWIs targets, eleven facilities (3.6%) met five targets, 53 (17.4%) facilities met four targets, 97 (31.9%) met three targets, 107 (35.2%) met two targets and 35 (11.5%) facilities met only one target. The facility which met all the 6 EWI targets was Mbarara Regional Referral Hospital, which is a centre of excellence, as well as a teaching University Hospital. Twenty eight (80.0%) of the 35 facilities which met only the indicator on dispensing practices were health centres.

## Discussion

This report, provides insight into specific programme factors associated with increased risk of HIVDR, and will inform interventions designed towards improvement of ART service delivery in Uganda. Several factors are associated with the emergence of HIVDR including; viral factors (e.g. subtype, replication capacity, and pre-existing polymorphisms); drug-related factors (e.g. drug potency, pharmacokinetics, drug-drug interactions, drug tolerability, and genetic barrier to selection of resistance); and programme factors (e.g. patient adherence to prescribed ART, drug supply continuity, and retention of patients on treatment) [8]. Whereas viral and drug-related factors cannot be controlled at program level, most causes of drug resistance are preventable and are within the control of public health or programme action [8].

*On-time pill pick-up* is an objective measure of population adherence to ART and is associated with LTFU, virological failure, HIVDR and increased mortality [14, 15]. In Uganda, adherence is monitored at each patient visit and measured by; VL monitoring, patient self—reporting and by pill counting in which a patient is awarded a score of either good, fair or poor [11]. Data on this indicator was incomplete with patient follow up data missing in some sites. This could have been routinely collected but not recorded on patient ART cards. Amongst the patients assessed, the pill pick-up indicator target did not meet the WHO-recommendation. In comparison to other countries in the continent; a nationally representative survey conducted in Namibia among 193 sites in 2014, showed that 81.9% (CI: 81.1–82.8) of the patients met this indicator, whereas 21% of the clinics achieved excellent performance in this indicator [16]. Cameroon in 2013 reported, 33.3% (n = 12) of the clinics assessed met the target [17]. The 2016 Global Report for EWI for HIVDR, showed no statistically significant difference between clinic performance during this survey and results pooled from 438 clinics from 4 African countries, 69.9% (CI: 55.0–81.1). However, south east Asia recorded much better performance in this indicator, 91.4% (CI: 84.9–95.2) [8]. These findings show that there is a challenge of late ARV pick-up in Uganda and in sub Saharan Africa; and therefore the importance of designing programmatic interventions to monitor and optimize patient adherence to ART, in order to avoid preventable HIVDR.

*Retention on ART at 12 months* is important for optimal clinical outcomes in patients with HIV infection. In low and middle-income countries (LMIC), it has been demonstrated that most attrition and deaths occurred within the first 2 months of ART [18, 19]. It is therefore necessary that ART programs maintain high level of engagement with patients in care, and ensure continuous ART. Results from this survey, were similar to aggregated results from EWI surveys that showed lower rates of retention were observed amongst clinics in the African Region 67.6% (95% CI: 60.7–73.8%) when compared to all other regions combined 81.0% (95% CI: 77.3–84.3%) [8]; although no individual region had an average retention that exceeded the WHO-suggested target of 85%. This variation could be attributed to the higher gross national income in countries from Europe and America, whose ART programs are more equipped to follow up patients. In Uganda, like in many African countries, there is no unique identifier for HIV patients posing a challenge in tracking self-transfers to other facilities. The scale-up of ART provision to lower-level facilities, which was earlier limited to the urban referral facilities, has made ART more accessible. There is a possibility that some patients who are recorded as lost to follow-up (LTFU), are receiving ART from other facilities or are actually dead [20, 21]. Therefore, attrition of patients at facilities, may not necessarily reflect treatment cessation in HIV patients.

*Dispensing Practices*: Inappropriate ARV combinations have been demonstrated to cause HIVDR [6]. None of the patient records reviewed revealed patients prescribed mono nor dual therapy at all facilities. This is attributed to the fixed-dose combinations of antiretrovirals where multiple antiretroviral drugs combined into a single pill.

*Pharmacy Stock-outs*: Interruption of ART can lead to emergence of HIVDR, therefore procurement of ARV drugs and a robust supply chain management within countries are critical to maintaining populations on ART [3, 14]. During this assessment, very few facilities did not experience a stock out. This low rate could be attributed to, poor drug procurement and supply management systems, resulting from inappropriate forecasting and quantification at facility level. This is further compounded by the implementation of the ‘test and treat’ policy in Uganda in 2017 that recommended all people tested HIV-positive to start treatment immediately leading to increase in ART enrolment [10, 22]. Despite the introduction of a web-based ARV ordering system, there still remains a need to train of dispensers and pharmacists at health facilities in the use of this system. Most of the facilities affected were lower level health centres. This could be attributed to the challenges in the supply chain for essential drugs in Uganda, which employs a mixed "push" and "pull" system in which upper-level health facilities order drugs, resulting in rationalization of supplies to lower level facilities.

Zakumupa et al in their appraisal of the ARV supply chain system in Uganda [22, 23], observed that inaccurate ART medicine quantification, untimely ARV orders,over stocking of select ARVs, maldistribution of drugs contributed to stock-outs of drugs at facilities. In addition, Windisch noted that poor quality and inefficiencies in the drug supply chain management in Uganda was caused by parallel processes and information systems amplified by inadequacies at all levels of the health system, including the areas of financing, governance, human resources and information [23]. The challenge of stock-outs is not limited to only Uganda; in other sub-Saharan countries, performance for this indicator ranged between 0% - 69% [8, 16, 24, 25] which was lower than the Asian/ Caribbean and European counterparts [8, 26, 27]. There is therefore need to improve the national procurement and supply chain systems and to ensure last-mile distribution of ARVs in order to have continuous availability of ARVs to patients.

*Viral load suppression*: The association between virological failure and HIVDR is strong, therefore achieving high levels of VS reduces morbidity and mortality and decreases HIV incidence. The prevalence of VS was below the WHO target both at patient and at facility level, therefore more effort needs to be directed towards achieving the UNAIDS target of 90% of people on ART achieving VL suppression. Viral load monitoring should also be used as a standard of care, whereby patients who are not suppressing are targeted for more clinical monitoring.

*Viral load completion*: The VL completion indicator evaluates a programme’s capacity to implement VL testing and assure that the result is returned to a patient’s records, maximizing the likelihood of it being reviewed and acted upon by providers, if necessary [8]. Only half of the facilities met this indicator, and in 7 facilities, no VL patient records were found. As much as the Uganda ART program has ensured universal VL testing, which is free of charge, and provided free transportation of specimen to the national VL testing laboratory through the national specimen transportation network, there still remains a need to expand testing to all health facilities and creation of awareness among clinicians to ensure all eligible patients conduct VL testing. Some facilities especially privately owned, were not linked to the national specimen transportation network, and so, were not conducting routine VL testing for their patients. In many facilities, VL results are kept in a separate file from the patient’s clinical records. There is therefore a possibility of some patients having conducted VL testing but with results unavailable in their patient records, hence the low performance in this indicator.

Low performance of facilities in most of the indicators is concerning with only one facility meeting all the indicators. Mbarara Regional Referral Hospital, is a centre of excellence, a teaching hospital for Mbarara University of Science and Technology from western Uganda. Most of the facilities which met only one indicator are health centres which do not have physicians running their ART clinics, and are also understaffed.

Despite being able to collect all indicators in the protocol as planned during this survey, implementing this assessment was not without

### Limitations

Four of the targeted facilities did not contribute to this report as two were in hard-to-reach terrain, and three others did not have patient records readily available. This however did not affect the power of the survey, as non-response was accounted for by facilities that exceeded the minimum sample size for patients, especially sites with electronic medical record where all eligible patients were considered. In addition, bivariable analysis was conducted without any adjustments for potential confounders. Also, although public facilities in Uganda account for more 62% of HIV service delivery in Uganda, the private-for-profit sector in ART provision represented a small proportion.

In conclusion, there still remains a challenge to long-term ART programmatic success in maintaining populations of patients on ART without treatment interruptions which may arise due to poor patient adherence and also which may occur as a consequence of ARV supply interruption at ART facilities. It is therefore of high importance that the national ART program ensures intensified follow-up for patients, continuous uninterrupted supply of ARV drugs at all facilities and VL monitoring of all patients on ART in order to improve patient outcomes and avert preventable HIVDR. The need for a national medical records system which integrates socio-demographic, clinical, lab and all other patient information, cannot be over emphasized. The study findings point to the need for further qualitative interrogation of some of the study findings including understanding of patient-level reasons for poor ART adherence, reasons for drug stock-outs and understanding poor viral load coverage, despite having access to the national specimen transportation network.

Monitoring of HIVDR EWIs should therefore be scaled up in the country and integrated into routine practice.

## Supporting information

S1 Data (XLSX)
